# *NLRC* and *NLRX* gene family mRNA expression and prognostic value in hepatocellular carcinoma

**DOI:** 10.1002/cam4.1202

**Published:** 2017-09-29

**Authors:** Xiangkun Wang, Chengkun Yang, Xiwen Liao, Chuangye Han, Tingdong Yu, Ketuan Huang, Long Yu, Wei Qin, Guangzhi Zhu, Hao Su, Xiaoguang Liu, Xinping Ye, Bin Chen, Minhao Peng, Tao Peng

**Affiliations:** ^1^ Department of Hepatobiliary Surgery The First Affiliated Hospital of Guangxi Medical University Nanning Guangxi Province 530021 China; ^2^ Department of Hepatobiliary and Pancreatic Surgery The First Affiliated Hospital of Zhengzhou University Zhengzhou Henan Province 450000 China; ^3^ Department of Hepatobiliary Surgery Affiliated Hospital of Guangdong Medical University Zhanjiang Guangdong Province 524001 China

**Keywords:** mRNA expression, NLRC, NLRX, hepatocellular, carcinoma, prognosis

## Abstract

Nucleotide‐binding oligomerization domain (NOD)‐like receptor (NLR)C and NLRX family proteins play a key role in the innate immune response. The relationship between these proteins and hepatocellular carcinoma (HCC) remains unclear. This study investigated the prognostic significance of NLRC and NLRX family protein levels in HCC patients. Data from 360 HCC patients in The Cancer Genome Atlas database and 231 patients in the Gene Expression Omnibus database were analyzed. Kaplan–Meier analysis and a Cox regression model were used to determine median survival time (MST) and overall and recurrence‐free survival by calculating the hazard ratio (HR) and 95% confidence interval (CI). High *NOD2* and low *NLRX1* expression in tumor tissue was associated with short MST (P = 0.012 and 0.014, respectively). A joint‐effects analysis of *NOD2* and *NLRX1* combined revealed that groups III and IV had reduced risk of death from HCC as compared to group I (adjusted P = 0.001, adjusted HR = 0.31, 95% CI = 0.16–0.61 and adjusted P = 0.043, adjusted HR = 0.63, 95%CI = 0.41–0.99, respectively). *NOD2* and *NLRX1* expression levels are potential prognostic markers in HCC following hepatectomy.

## Introduction

Hepatocellular carcinoma (HCC) is the most common type of liver cancer and the fifth most common malignancy worldwide, ranking as the third leading cause of cancer‐related death 1. The 5‐year relative survival rate for HCC is approximately 7% 1. About half of the 782,500 liver cancer cases newly diagnosed worldwide in 2012 were in China 2, 3. Infection with hepatitis B and C viruses (HBV and HCV, respectively) is the major cause of hepatocarcinogenesis 4. Other risk factors include cirrhosis, aflatoxin exposure, hemochromatosis, obesity, diabetes mellitus, and metabolic factors 4. In addition, the high frequency of late‐stage disease, metastasis, de novo tumor formation in the diseased liver 5, high rate of recurrence 6, and aberrant gene expression 7, 8 contribute to poor patient prognosis.

The dysregulation of various genes has been linked to HCC prognosis 9, 10. We hypothesized that certain gene families are associated with HCC prognosis; a literature search revealed that only few have been identified 11, 12. Nucleotide‐binding oligomerization domain (NOD)‐like receptors (NLRs) are cystosolic pattern recognition receptors (PRRs) and include five subfamilies—that is, NLRA, NLRB, NLRC, NLRP, and NLRX. These receptors play an important role in monitoring the intracellular microenvironment and mediating inflammation and pathogen clearance 13. The NLRC family has five members—that is, *NOD1*,* NOD2*,* NLRC3*,* NLRC4*, and *NLRC5* 13. *NOD1* and *NOD2* are important components of the innate immune system that protects organisms from *Helicobacter pylori* infection 14 and function as pattern‐recognition molecules that initiate intracellular signaling pathways in response to pathogen‐associated molecular patterns 15. *NLRC3* was identified as a negative regulator of type I interferon and proinflammatory cytokine production 16. In contrast, the functions of *NLRC4* are not well understood 17. *NLRC5* is negative regulator of nuclear factor *κ*B and type I interferon pathways, and is thus important for innate immune system homeostasis 18. *NLRX1*, the only NLR localized in mitochondria and the sole member of the NLRX family, was found to stimulate reactive oxygen species production following *Shigella flexneri* infection 19.

Abnormal inflammation is considered as an indicator of tumorigenesis and malignancy. Four major families of PRR—that is, toll‐like receptors (TLRs), C‐type lectin receptors, RIG‐I‐like receptors, and NLRs—have been implicated in cell proliferation, angiogenesis, tissue remodeling and repair, and tumorigenesis 20. Most studies of PRR signaling in malignancies to date have focused on TLR family members. However, recent studies indicate that NLR family members play a direct or indirect role in cancer cell death, angiogenesis, invasion, and metastasis 21, 22. The present study investigated the prognostic value of NLRC and NLRX family proteins in HCC.

## Material and Methods

### Patient information

We used an online resource (http://merav.wi.mit.edu/; accessed February 10, 2017) to identify genes of the NLRC and NLRX families that are differentially expressed between normal liver tissue and primary liver tumors. We then used the online website (http://www.oncolnc.org/; accessed September 2, 2017) and The Cancer Genome Atlas (TCGA), (http://tcga-data.nci.nih.gov/tcga) to obtain information on mRNA expression levels of *NOD1*,* NOD2*,* NLRC3*,* NLRC4*,* NLRC5*, and *NLRX1* at a 75% cutoff; the results presented here are based in part on data generated by TCGA Research (http://cancergenome.nih.gov/) 23. Clinical data of 360 patients were also downloaded, including race, gender, age, body mass index (BMI), tumor‐node‐metastasis (TNM) stage, survival time (days), and survival status.

Gene expression profiles were obtained from an independent dataset (GSE14520) in the National Center for Biotechnology Information Gene Expression Omnibus (GEO) (https://www.ncbi.nlm.nih.gov/geo/query/acc.cgi?acc=GSE14520, accessed February 15, 2017) database 24. The dataset contained expression profiles generated from [HT_HG‐U133A] Affymetrix HT Human Genome U133A 24 and [HT_HG‐U133A_2] Affymetrix HT Human Genome U133A_2.0 25 arrays. To avoid a batch effect, we selected a profile from the former array that had more patients (*n* = 231 HCC patients) than the latter. Furthermore, the GeneMANIA website (http://genemania.org/; accessed February 18, 2017) was used to analyze interaction networks of the two NLR families 26.

### Functional enrichment analysis of NLRC and NLRX families

The Database for Annotation, Visualization, and Integrated Discovery (DAVID) v.6.7 (https://david-d.ncifcrf.gov/, accessed February 25, 2017) 27, 28 was used for functional enrichment analyses, including gene ontology (GO) functional analysis and Kyoto Encyclopedia of Genes and Genomes (KEGG) pathway analysis. The former included biological process (BP) and molecular function (MF) terms; in the latter, no results were returned for NLRC and NLRX families.

### Survival analysis

In TCGA database, mRNA expression levels in 360 HCC patients were divided into two groups at a cutoff value of 75%; low and high expression groups comprised 270 and 90 patients, respectively. The same cutoff value was applied to the GEO database in order to ensure a reasonable comparison between the two databases. Median survival time (MST) was used to evaluate the prognosis of HCC patients in TCGA database, whereas overall survival (OS) and recurrence‐free survival (RFS) were used to assess that of patients in the GEO database. Sex, age, and TNM stage were adjusted in the Cox proportional hazards regression model in TCGA database, whereas gender, age, HBV infection status, alanine aminotransferase (ALT) status, main tumor size, multinodule status, cirrhosis, alphafetoprotein (AFP) level, and Barcelona Clinic Liver Cancer (BCLC) stage were adjusted in the Cox proportional hazards regression model in the GEO database.

### Joint‐effects analysis

Only *NOD2* and *NLRX1* were statistically significant in TCGA database. We carried out a joint‐effects analysis of the combination of *NOD2* and *NLRX1*.

The combination of *NOD2* and *NLRX1* included group I (high *NOD2* and low *NLRX1* expression), group II (high *NOD2* and high *NLRX1* expression), group III (low *NOD2* and high *NLRX1* expression), and group IV (low *NOD2* and low *NLRX1* expression).

Sex, age, and TNM stage were adjusted in the Cox proportional hazards regression model according to the combination of genes in TCGA database.

### Statistical analysis

Pearson correlation coefficients were used to assess correlations among *NOD1*,* NOD2*,* NLRC3*,* NLRC4*,* NLRC5*, and *NLRX1* genes. Kaplan–Meier survival analysis and the log‐rank test were used to calculate MSTs and *P* values. Uni‐ and multivariate survival analyses were performed using the Cox proportional hazards regression model. Hazard ratios (HRs) and 95% confidence intervals (CIs) were calculated with the Cox proportional hazards regression model with adjustment for influential clinical characteristics such as gender, age, HBV infection status, ALT status, main tumor size, multinodule status, cirrhosis, TNM stage, and AFP level. *P* < 0.05 was considered as statistically significant. Vertical scatter plots and survival curves were plotted using GraphPad Prism v.5.0 (La Jolla, CA). Statistical analyses was performed with SPSS software v.22.0 (IBM, Chicago, IL).

## Results

### Characteristics of patients in TCGA and GEO databases

Detailed characteristics of the 360 patients in TCGA are shown in Table 1. Race, gender, age, BMI, were not associated with MST. On the other hand, TNM stage, *NOD2* and *NLRX1* levels showed significant associations with MST (P <0.001; adjusted P= 0.014 and 0.011, respectively).

**Table 1:** Demography and clinical characteristics of 360 HCC patients in TCGA database

Variables	Patients (n = 360)	No. of events (%)	MST (moths)	HR (95% CI)	Log‐rank *P*
Race					0.176
Asian	155	44 (28.4%)	NA	Ref.	
White+others	196	78 (39.8%)	47	1.29 (0.89–1.88)	
Missing^Đ^	9				
Gender					0.311
Male	244	78 (32.0%)	83	Ref.	
Female	116	48 (41.4%)	52	1.21 (0.84–1.73)	
Age(year)					0.362
<60	168	54 (32.1%)	84	Ref.	
≥60	189	70 (37.0%)	56	1.18 (0.83–1.68)	
Missing^†^	3				
BMI					0.496
≤25	193	66 (34.2%)	82	Ref.	
>25	137	45 (32.8%)	71	0.88 (0.60–1.28)	
Missing^ý^	30				
TNM stage					**<0.001**
A+B	252	66 (26.2%)	84	Ref.	
C+D	87	48 (55.2%)	26	2.48 (1.71–3.61)	
Missing^Ĺ^	21				.
*NOD1*					0.197
Low	270	89 (33.0%)	71	Ref	
High	90	37 (41.1%)	50	1.29 (0.88–1.89)	
*NOD2*					**0.012**
Low	270	82 (30.4%)	83	Ref	
High	90	44 (48.9%)	47	1.60 (1.11–2.30)	
*NLRC3*					**0.043**
Low	270	103 (38.1%)	54	Ref	
High	90	23 (25.6%)	82	0.63 (0.40–0.99)	
*NLRC4*					0.700
Low	270	92 (34.1%)	60	Ref.	
High	90	34 (37.8%)	56	1.08 (0.73–1.60)	
*NLRC5*					0.277
Low	270	98 (36.3%)	56	Ref.	
High	90	28 (31.1%)	60	0.79 (0.52–1.21)	
*NLRX1*					**0.015**
Low	270	103 (38.1%)	52	Ref.	
High	90	23 (25.6%)	85	0.57 (0.36–0.90)	

BMI, body mass index; TNM stage, tumor, node and metastasis stage; MST, median survival time; HR, hazard ratio; 95% CI, 95% confidence interval; Ref, reference; *NOD*,=nucleotide‐binding oligomerization domain; *NLRC=* nucleotide‐binding oligomerization domain‐like receptors family CARD domain containing; *NLRX1*, nucleotide‐binding oligomerization domain‐like receptors family member X1; Missing^Đ^, information of race was unavailable in 9 patients; Missing^†^,information of age was unavailable in 3 patients; Missing^ý^, information of BMI was unavailable in 30 patients; Missing^Ĺ^, information of TNM stage was unavailable in 21 patients.

Bold value in all the tables were statistically significant (*P* ≤ 0.05).

The characteristics of the 231 patients in the GEO database are shown in Table 2. Sex, main tumor size, multinodule status, cirrhosis, BCLC stage, and AFP level were significantly associated with OS (*P *= 0.048, <0.001, 0.003, 0.002, 0.001, and 0.001, respectively), whereas gender, main tumor size, cirrhosis, and BCLC stage were significantly associated with RFS (*P *= 0.001, 0.019, 0.016, and <0.001, respectively).

**Table 2:** Demography and clinical characteristics of 231 HCC patients in GEO database

Variables	Patients (*n* = 231)	Overall survival			Recurrence‐free survival		
		MST (months)	HR (95%CI)	Log‐rank *P*	MST (months)	HR (95%CI)	Log‐rank *P*
Gender				**0.048**			**0.001**
Male	191	NA	Ref.		40	Ref.	
Female	30	NA	0.59 (0.34–1.00)		NA	0.47 (0.29–0.75)	
Missing^Ʒ^	10						
Age				0.852			0.937
≤60	181	NA	Ref.		46	Ref.	
>60	40	NA	0.96 (0.65–1.44)		37	1.01 (0.73–1.41)	
Missing^Ʒ^	10						
HBV‐virus status				0.147			0.090
AVR‐CC	56	NA	Ref.		30	Ref.	
CC+NO	162	NA	0.80 (0.56–1.09)		48	0.78 (0.59–1.04)	
Missing^ƛ^	13						
ALT				0.710			0.088
≤50U/L	130	NA	Ref.		53	Ref.	
>50U/L	91	NA	1.06 (0.78–1.44)		40	1.25 (0.97–1.61)	
Missing^Ʒ^	10						
Main tumor size				**<0.001**			**0.019**
≤5 cm	140	NA	Ref.		51	Ref.	
>5 cm	80	53	1.87 (1.38–2.55)		30	1.37 (1.05–1.78)	
Missing^ƥ^	11						
Multinodular				**0.003**			0.135
Yes	45	48	Ref.		27	Ref.	
No	176	NA	0.59 (0.42–0.84)		49	0.79 (0.58–1.08)	
Missing^Ʒ^	10						
Cirrhosis				**0.002**			**0.016**
Yes	203	NA	Ref.		38	Ref.	
No	18	NA	0.23 (0.09–0.63)		NA	0.50 (0.28–0.89)	
Missing^Ʒ^	10						
BCLC stage				**<0.001**			**<0.001**
0+A	168	NA	Ref.		58	Ref.	
B+C	51	20	3.68 (2.66–5.06)		18	2.84 (2.14–3.77)	
Missing^Ɯ^	12						
AFP				**0.001**			0.093
≤300 ng/ml	100	NA	Ref.		49	Ref.	
>300 ng/ml	118	NA	0.60 (0.44–0.81)		31	0.80 (0.62–1.04)	
Missing^ƛ^	13						
NOD1				0.862			0.379
Low	187	NA	Ref.		42	Ref.	
High	44	NA	0.97 (0.69–1.37)		53	0.88 (0.65–1.18)	
NOD2				0.262			0.449
Low	169	NA	Ref.		46	Ref.	
High	62	NA	1.21 (0.86–1.70)		40	1.12 (0.84–1.50)	
NLRX1				0.114			0.894
Low	168	NA	Ref.		46	Ref.	
High	63	NA	0.74 (0.51–1.08)		43	1.02 (0.76–1.37)	

AVR‐CC, active viral replication chronic carrier; CC, chronic carrier; ALT, alanine aminotransferase; AFP, alpha fetoprotein; BCLC stage, Barcelona Clinic Liver Cancer; Missing^Ʒ^, information of gender, age, ALT, multinodular, cirrhosis was unavailable in 10 patients; Missing^ƥ^, information of main tumor size was unavailable in 11 patients; Missing^Ɯ^, information of BCLC stage was unavailable in 12 patients; Missing^ƛ^, information of HBV‐virus status and AFP was unavailable in 13 patients.

Bold value in all the tables were statistically significant (P≤0.05).

### Correlation analysis of NLRC and NLRX family mRNA expression levels in TCGA and GEO databases

**Figure 1:**
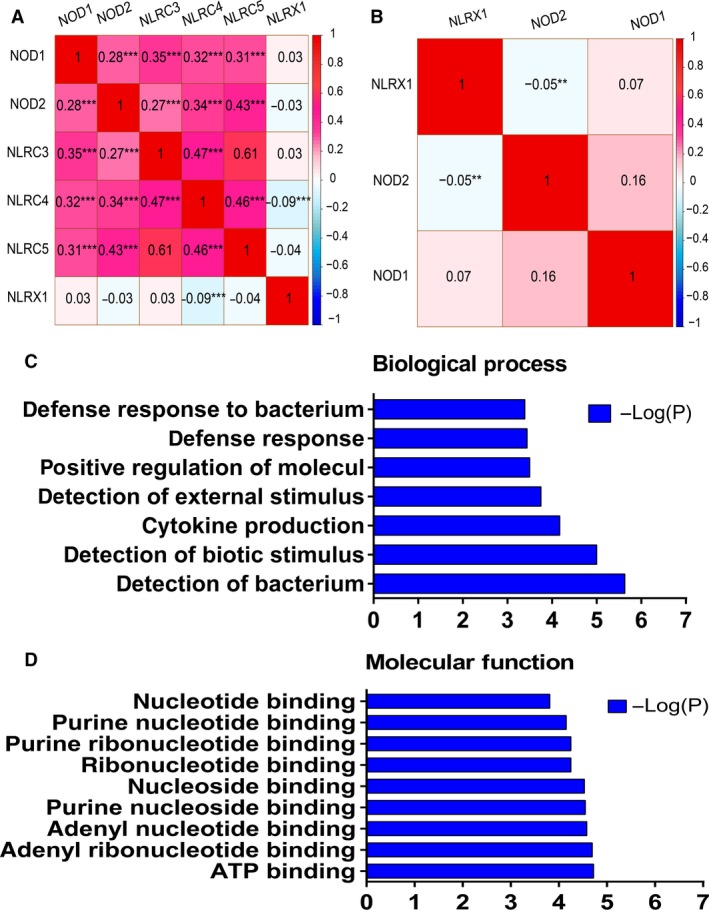
Matrix graphs of Pearson's correlations of *NOD1*,*NOD2*,*NLRC3*,*NLRC4*,*NLRC5*, and *NLRX1* gene expression levels in TCGA and GEO databases and analysis of GO terms enriched in NLRC and NLRX families performed using DAVID. (A) Genes expression levels in the TCGA database. (B) Gene expression levels in the GEO database. (C) GO terms for biological processes. (D) GO terms for molecular function. *^*^ *P* < 0.01, *^**^ *P* < 0.001.

We calculated Pearson correlation coefficients between NLRC and NLRX families. In TCGA database, *NOD1* was correlated with other NLRC family members (all *P* < 0.001) but not with the NLRX family member (*P *= 0.541), except for *NRLC4* (*P* < 0.001, *r *= −0.09) (Fig. 1A). Only *NOD1*,* NOD2*, and *NLRX1* expression data were available in the GEO database. *NOD1* was correlated with *NOD2* (*P *= 0.001) but not with the NLRX family member (*P *= 0.164); there was also no correlation between *NOD2* and the NLRX family member (*P *= 0.341) (Fig. 1B).

### GO functional annotation analysis of NLRC and NLRX families

To investigate biological functions of the NLRC and NLRX families, BP and MF were evaluated in the GO analysis (Fig. 1C and D). In the KEGG pathway analysis, DAVID did not identify any associations between NLRC and NLRX families.

### Survival analysis of NLRC and NLRX family mRNA expression levels in TCGA and GEO databases

The characteristics of patients in TCGA database related to prognosis including age, gender, and TNM stage were analyzed with a multivariate Cox proportional hazards regression model. *NOD2* and *NLRX1* showed significant associations with MST (adjusted P = 0.014, adjusted HR = 1.64, 95% CI = 1.11–2.44; adjusted P = 0.011, adjusted HR = 0.53, 95% CI = 0.33–0.86, respectively) (Table 3). For patients in the GEO database, characteristics such as gender, age, HBV viral infection status, ALT status, main tumor size, multinodule status, cirrhosis, AFP level, and BCLC stage were analyzed with a multivariate Cox proportional hazards regression model. *NOD1*,* NOD2*, and *NLRX1* were not significantly associated with OS or RFS (Table 4).

**Table 3:** Prognostic survival analysis of *NOD1, NOD2, NLRC3, NLRC4, NLRC5* and *NLRX1* in TCGA database

Gene	Patients (n = 360)	MST (months)	Crude HR (95%CI)	Crude *P*	Adjusted HR^§^ (95%CI)	Adjusted *P* ^§^
*NOD1*						
Low	270	71	Ref	0.197	Ref.	0.183
High	90	50	1.29 (0.88–1.89)		1.32 (0.88–1.97)	
*NOD2*						
Low	270	83	Ref.	**0.012**	Ref.	**0.014**
High	90	47	1.60 (1.11–2.30)		1.64 (1.11–2.44)	
*NLRC3*						
Low	270	54	Ref.	**0.043**	Ref.	0.207
High	90	82	0.63 (0.40–0.99)		0.74 (0.46–1.19)	
*NLRC4*						
Low	270	60	Ref.	0.700	Ref.	0.461
High	90	56	1.08 (0.73–1.60)		1.17 (0.77–1.79)	
*NLRC5*						
Low	270	56	Ref.	0.277	Ref.	0.168
High	90	60	0.79 (0.52–1.21)		0.73 (0.47–1.14)	
*NLRX1*						
Low	270	52	Ref.	**0.015**	Ref.	**0.011**
High	90	85	0.57 (0.36–0.90)		0.53 (0.33–0.86)	

Bold value in all the tables were statistically significant (*P* ≤ 0.05).

**Table 4:** Prognostic survival analysis of *NOD1, NOD2,* and *NLRX1* in GEO database

Gene	Patients	Overall survival				Recurrence‐free survival			
	(*n* = 231)	Crude HR (95% CI)	Crude *P*	Adjusted HR (95%CI)	Adjusted *P*	Crude HR (95%CI)	Crude *P*	Adjusted HR^a^(95%CI)	Adjusted*P* a
NOD1			0.862		0.210		0.379		0.051
Low	187	Ref.		Ref.		Ref.		Ref.	
High	44	0.97 (0.69–1.37)		0.79 (0.55–1.14)		0.88 (0.65–1.18)		0.74(0.54–1.00)	
NOD2			0.262		0.390		0.449		0.759
Low	169	Ref.		Ref.		Ref.		Ref.	
High	62	1.21 (0.86–1.71)		1.17 (0.82–1.65)		1.12 (0.84–1.50)		1.05 (0.78–1.41)	
NLRX1			0.114		0.056		0.894		0.768
Low	168	Ref.		Ref.		Ref.		Ref.	
High	63	0.74 (0.51–1.08)		0.68 (0.46–1.01)		1.02 (0.76–1.37)		0.96 (0.71–1.29)	

a Adjusted *P*, adjustment of gender, age, HBV‐virus status, ALT, main tumor size, multinodular, cirrhosis, AFP, and BCLC stage.

### Analysis of mRNA expression levels in TCGA and GEO databases

**Figure 2:**
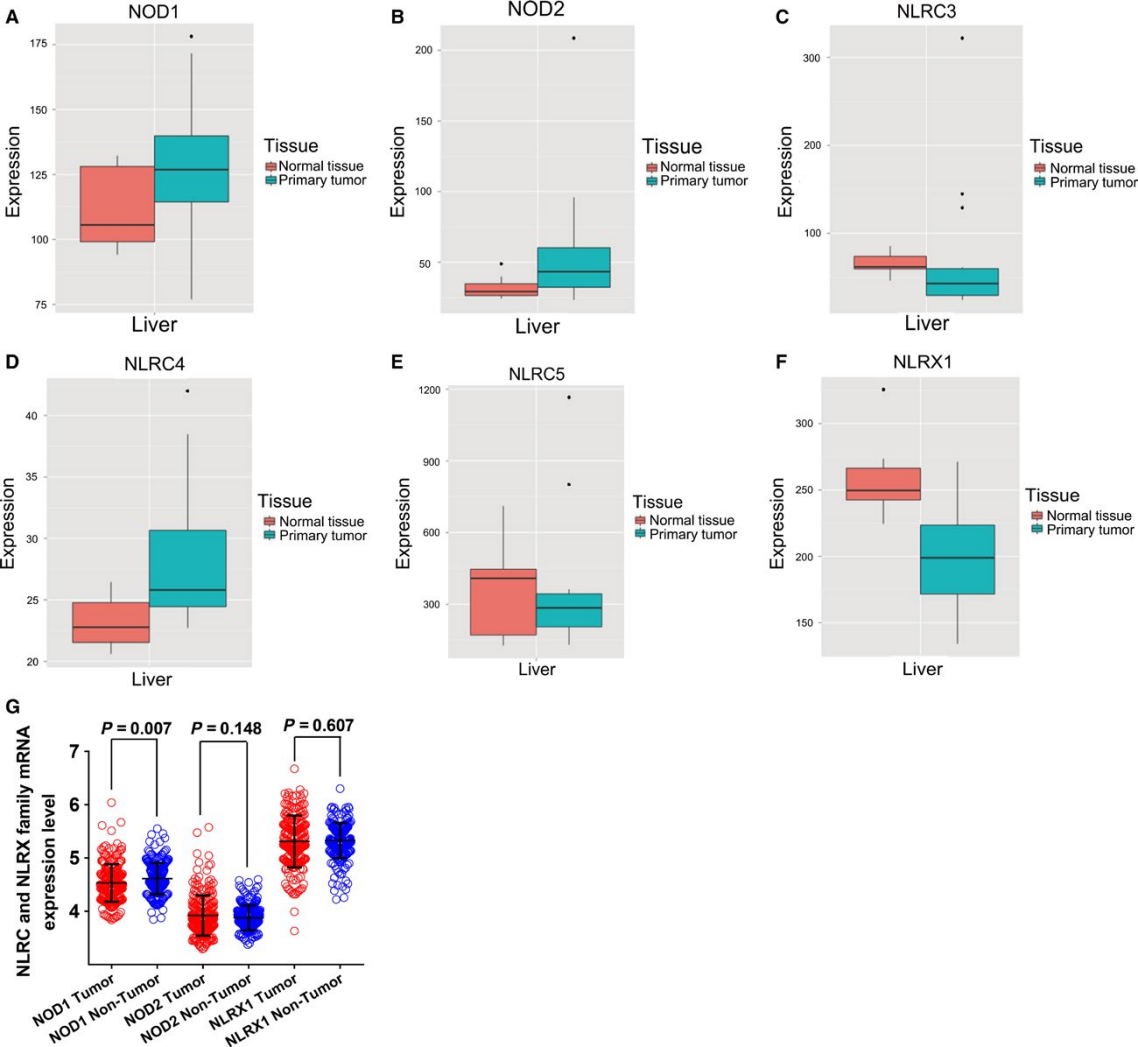
(A–F) mRNA expression levels of *NOD1* (A), *NOD2* (B), *NLRC3* (C), *NLRC4* (D), *NLRC5* (E), and *NLRX1* (F) genes in normal liver tissue and primary liver tumors. G, *NOD1*,*NOD2*, and *NLRX1* genes in the GEO database.

Box plots of the expression levels of six genes were downloaded from an online website (Fig. 2A–F). *NLRC3*,* NLRC5*, and *NLRX1* were highly expressed in normal liver tissue whereas the expression in primary liver tumors was low. Scatter plots of *NOD1*,* NOD2*, and *NLRX1* mRNA expression level in the GEO database revealed that only *NOD1* expression differed significantly between tumor and nontumor tissue (*P *= 0.007; Fig. 2G).

**Figure 3:**
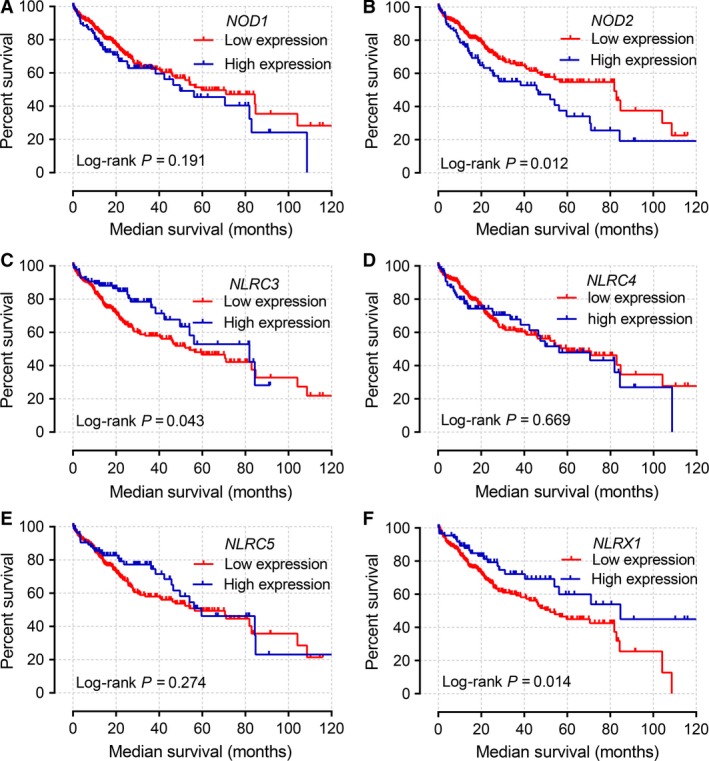
(A–F) Kaplan–Meier survival curves of *NOD1* (A), *NOD2* (B), *NLRC3* (C), *NLRC4* (D), *NLRC5* (E), and *NLRX1* (F) genes in the TCGA database. MST was stratified by the above‐listed genes.

Kaplan–Meier curves of mRNA expression levels in TCGA database at a cutoff of 75% are shown in Figure 3. *NOD2*,* NLRC3*, and *NLRX1* all had significant *P* values at this cutoff value (*P *= 0.011, 0.043, and 0.014, respectively).

**Figure 4:**
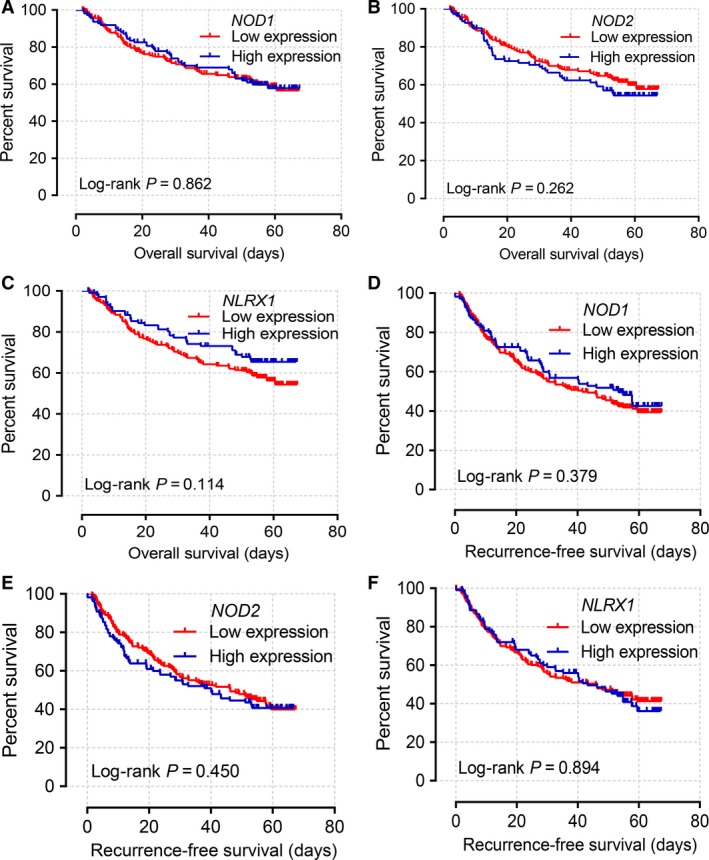
(A–F) Kaplan–Meier survival curves of OS (A–C) and RFS (D–F) stratified by *NOD1* (A, D) *NOD2* (B, E), and *NLRX1* (C, F) genes in the GEO database.

**Figure 5:**
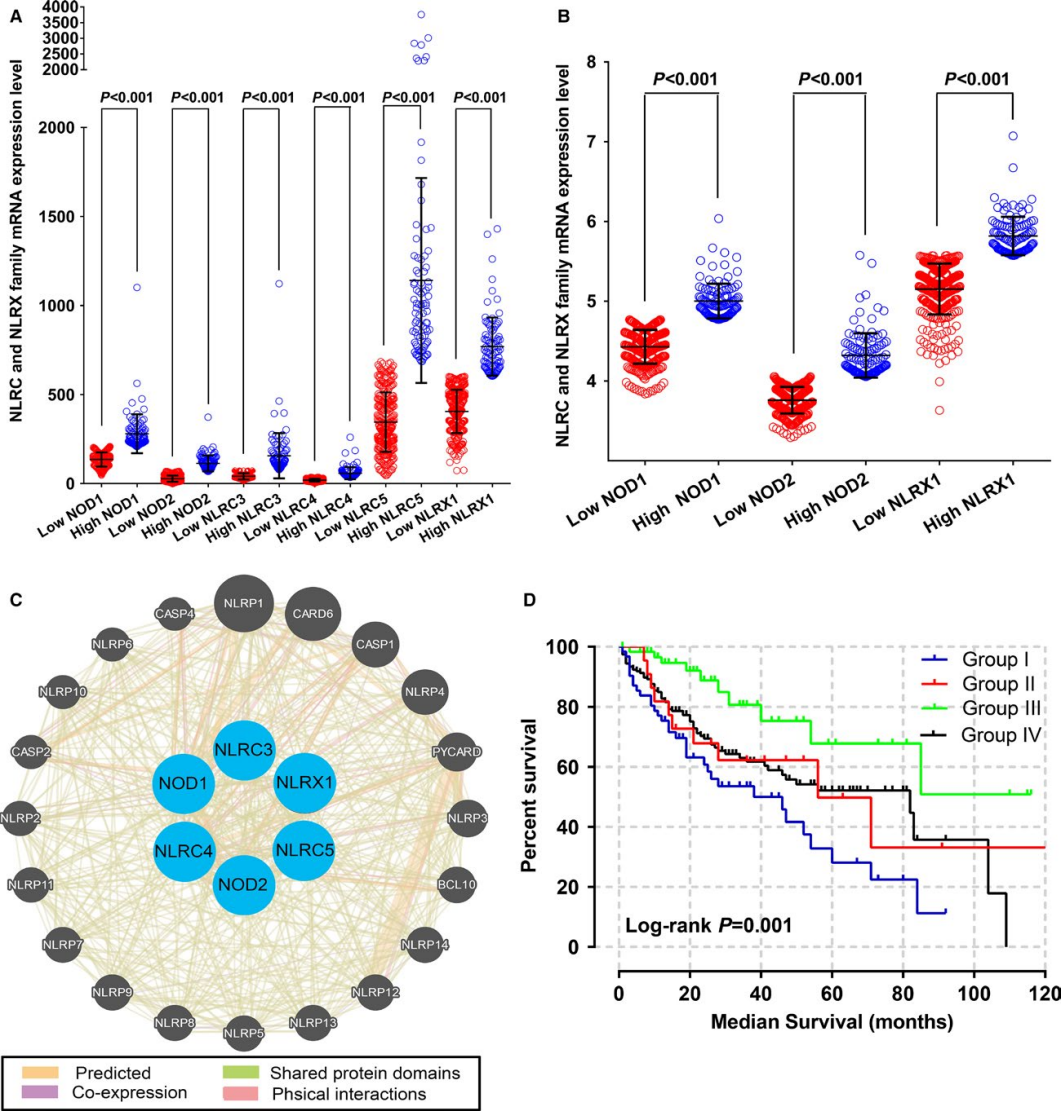
Scatter plots of *NOD1*,* NOD2*,* NLRC3*,* NLRC4*,* NLRC5*, and *NLRX1* gene expression levels in TCGA (A), GEO (B) databases and gene–gene interaction networks among selected genes constructed by GeneMANIA (C) and survival curves for joint‐effects analysis of the combination of *NOD2* and *NLRX1* genes in TCGA database (D).

Kaplan–Meier curves of mRNA expression levels in the GEO database at 75% cutoff are shown in Figure 4. *NOD1*,* NOD2*, and *NLRX1* did not have significant *P* values for OS and RFS (all *P* > 0.05). Scatter plots of the expression levels of six genes in the TCGA and GEO databases at a 75% cutoff are shown in Figure 5A and B.

### Joint‐effects analysis of NLRC and NLRX family mRNA expression levels in TCGA database

We carried out a joint‐effects analysis for the combination of NOD2 and NLRX1. In the joint‐effects analysis of the combination of NOD2 and NLRX1, group I had the shortest MST of 38 months (adjusted P = 0.007), whereas group III had the longest MST of 85 months (adjusted P = 0.001, adjusted HR = 0.31, 95% CI = 0.16–0.61) (Table 5). Interaction networks among *NOD1, NOD2, NLRC4, NLRC5*, and *NLRX1* are shown in Figure 5C. Kaplan–Meier survival curves of the analyses of two genes are shown in Figures 5D.

**Table 5:** Joint‐effects analysis of the combination of *NOD2* and *NLRX1* in TCGA database

Group	*NOD2* expression	*NLRX1* expression	Patients (n = 360)	MST (months)	Crude *P*	Crude HR (95% CI)	Adjusted P*	Adjusted HR* (95% CI)
I	High	Low	67	38	**0.005**	Ref.	**0.007**	Ref.
II	High	High	23	56	0.142	0.59 (0.29–1.20)	0.228	1.61 (0.27–1.36)
III	Low	High	67	85	**0.001**	0.32 (0.17–0.62)	**0.001**	0.31 (0.16–0.61)
IV	Low	Low	203	82	**0.022**	0.62 (0.42–0.93)	**0.043**	0.63 (0.41–0.99)

Adjusted P*, adjustment for gender, age, TNM stage.

Bold value in all the tables were statistically significant (*P* ≤ 0.05).

## Discussion

In this study, we investigated the association between NLRC and NLRX family genes and HCC. We determined that the mRNA expression levels of these two NLR families are associated with distinct prognoses. Thus, the mRNA expression levels of NLRC and NLRX family genes alone or in combination—especially *NOD2*, and *NLRX1* combined—can predict HCC prognosis.

NLR family genes are known to regulate the formation of the inflammasome and pro‐inflammatory chemokines and cytokines that are involved in the host response to pathogens 29, 30. However, there is little known about the relationship between these gene families and cancer, especially HCC. *NOD1* is an important factor in the defense against *Pseudomonas aeruginosa* 31, *Listeria monocytogenes* 32, and *H. pylori* 33 infection and has been linked to Crohn's disease 34, 35, inflammatory bowel disease 36, and Behcet's disease 36. *NOD2* was found to be associated with Crohn's disease 37, ischemic cardiovascular disease 38, Blau syndrome 39, allergic rhinitis 40, and artherosclerosis 41. *NLRC3* is a biomarker for colorectal cancer 42; *NLRC4* was related to enterocolitis 43, recurrent macrophage activation syndrome 44, and familiar cold autoinflammatory syndrome 45; and *NLRC5* has been implicated in chronic periodontitis 46. *NLRX1* was found to be associated with risk of gastric cancer in the Chinese population 47. Interestingly, the other four genes in the *NLRC* and *NLRX* gene families did not show any direct or indirect associations with HCC, with the exception of *NOD1/NOD2* pathway, which acted synergistically with *NLRP3*.

In this study, we found that *NOD2* was highly expressed in primary liver tumors, which was associated with shorter MST. In contrast, *NLRX1* was expressed at low levels in primary liver tumors, which was also linked to short MST. In the joint‐effects analyses, groups I, had the shortest MST. In theory, the opposite trend in expression level for each gene should be associated with the best prognosis. Strikingly, this was only observed in group III.

AFP is a widely used serum diagnostic and prognostic biomarker for HCC. However, its prognostic value remains controversial. Serum AFP levels have been reported as an indicator of OS and RFS in HCC 48, 49. However, this was not confirmed in other studies 50, 51, 52. Its sensitivity for HCC screening ranges from 41 to 65% at a cutoff of 20 ng/mL 53, 54, 55, 56. In recent years, various biomarkers have emerged for diagnosing HCC and predicting patient outcome, including glypican 3 and insulin‐like growth factor (IGF)II mRNA 57, Keap1 and pNrf2 58, 3‐microRNA and AFP 59, CXCL1 60, minichromosome maintenance complex ‐7 61, and IGF1 receptor 62, among others.

Mitochondria release molecules such as cytochrome c and apoptosis‐inducing factor into the cytosol 63 and are associated with autophagy 64. Exogenous substances applied to HCC cell lines can affect the release of these molecules and thereby alter caspase‐independent apoptosis signaling (i.e., the mitochondrial pathway) 65. Mitochondrial NLRX1 expression is altered in liver tissue in HCC, suggesting that it could affect apoptosis in HCC, although the detailed mechanisms remain to be determined.

There were some limitations to our study that need to be recognized. Firstly, larger sample sizes are needed in order to increase the reliability of the findings. Secondly, more clinical data concerning tumor progression and prognosis such as smoking and drinking status, Child–Pugh scoring, presence of cirrhosis, transarterial chemoembolization, antitherapy status, radical resection status, pathological differentiation diagnosis, main tumor size, numbers of tumors, status of tumor capsules, regional invasion, intrahepatic metastasis, and vascular invasion should be included to better evaluate the relationship between the two NLR gene families and HCC. Thirdly, the more commonly used indices of OS and RFS should be applied to the evaluation of HCC prognosis. Fourth, further investigations focusing on functional part needs to be well explored in multi‐center, multi‐racial countries. And functional validation in a well‐designed clinical trial will be further studied in our future researches.

## Conclusion

Our study demonstrates that *NOD2,* and *NLRX1* may be potential prognostic biomarkers of HCC and their combination showed a strong interaction and better predictive value for HCC prognosis. Due to the small sample size and incomplete clinical information in this study, further well‐designed and larger sample size studies are necessary to validate our results.

## Conflict of Interest

None declared.
